# Effects of a Brief Strange Loop Task on Immediate Word Length Comparison: A Mindfulness Study on Non-striving

**DOI:** 10.3389/fpsyg.2019.02314

**Published:** 2019-10-11

**Authors:** Ying Hwa Kee, Khin Maung Aye, Raisyad Ferozd, Chunxiao Li

**Affiliations:** National Institute of Education, Nanyang Technological University, Singapore, Singapore

**Keywords:** mindfulness, paradox and ambiguity, reaction time, motivation, non-striving, goal setting

## Abstract

Non-striving is an important aspect of mindfulness practice, but it has not been sufficiently researched. This study examines whether a strange loop-based task – Infinite Water Scooping Task – performed for 10 min, has an effect on non-striving behavior and performance in a subsequent word length comparison task. Results showed that performance (number of correct trials) did not differ significantly between the two groups, though the experimental group tended to perform worse. However, participants in the experimental group took a significantly shorter time to respond to the word length comparison task than those in the control group. It is inferred that shorter time taken reflects response without investing much effort to count with care, i.e., non-striving. The present study demonstrates that the brief strange loop task implemented in this study elicited non-striving behavior compared to the effects of the control task, and this adds to the understanding of non-striving in the context of mindfulness. The Infinite Water Scooping Task may be useful for illustrating and teaching non-striving within mindfulness practice.

Mindfulness can be generally described as repetitive and sustained attentional efforts toward ongoing moments in an accepting and non-judgmental fashion (Kabat-Zinn, 1990). The consensus from previous operationalization of mindfulness points to attentional regulation and non-judgmental acceptance as two key aspects of mindfulness (Bishop et al., 2004; Lindsay and Creswell, 2017), consistent with the general definition above. The notion of acceptance and being non-judgmental underpins the uniqueness of mindfulness practice relative to other coping strategies, which are often focused on problem identification and intended eradication of problems, such as those found in traditional cognitive behavioral therapy approaches (Hofmann and Asmundson, 2008). In contract, the practice of acceptance and non-judgmental awareness within the mindfulness approach downplays problem eradication as mindfulness practice typically involves an observation mode, mere labeling of emotions, and distancing from mental content (Hinton et al., 2013). Suffice to say, the notion of “letting go” captures the sense of non-judgmental acceptance sufficiently well, as seen in previous literature (e.g., Frewen et al., 2008; Bergeron et al., 2016; Ruskin et al., 2017; Blackie and Kocovski, 2018). Thus, it can be assumed that implicit within the mindfulness approach is a sense of “letting go” or non-striving orientation which serves to obliterate the usual sense of resistance and judgmental stance toward undesired (or even desirable) circumstances and emotions.

Non-striving is an associated quality of mindfulness that is relatively less researched upon compared to other better known correlates such as compassion (Shonin et al., 2015), attention (Mak et al., 2018), and self-esteem (Randal et al., 2015). In English, the definition of striving is “to devote serious effort or energy” (Striving, n.d.). Accordingly, non-striving would mean the absence of devotion of serious effort or energy toward a task. In terms of mindfulness practice, an attitude of non-striving is about non-doing while undertaking the practice, trying less, and simply experiencing the moment (Kabat-Zinn, 1990). Kabat-Zinn (1990) elucidates that adopting a non-striving mind-set means that one is not desiring to change anything related to the moment through mindfulness practice, but instead being simply aware of the ongoing circumstances. This notion of non-striving discussed in secular mindfulness literature possibly roots from works in Eastern and Buddhist philosophy. Specifically, within the Chinese philosophy, the term *wu-wei* or effortless action has the connotation of non-striving as it refers to the harmonized state of mind while one performs actions spontaneously, with freedom from “the need for extended deliberation of inner struggle” (Slingerland, 2003, p. 7). This lack of inner struggle can be construed as non-striving. While discussion on non-striving remains scarce in the secular academic space today, mindfulness scholars such as Baer (2006) and Shapiro et al. (2018), echoing Kabat-Zinn’s view, too noted that mindfulness meditation should be practiced with no specific goal in mind, operationalizing the practical notion of non-striving for mindfulness practice to some extent. To further contribute to understanding of non-striving in the context of mindfulness practice, we conducted an experiment to investigate the effects of a brief strange loop task, purported to elicit sense of futility and paradoxity, on subsequent non-striving behavior and performance. We posit that this is an important endeavor that can help further operationalize the notion of non-striving for secular mindfulness practice.

The notion of non-striving in mindfulness practice is a paradox. Goal-oriented instructions for mindfulness practice, such as paying attention to the target object, are typically prescribed; and repeated attempts to adhere to the instruction are undertaken by the practitioner (Baer, 2006). But yet, mindfulness practice necessitates non-judgmental awareness, which could also, in theory, include openness and acceptance of whether there was success in adhering to the goal-oriented instruction. To illustrate, one may begin a mindfulness practice session with the goal of sitting for 30 minutes, with attention purposefully fixated on breathing and adopting a strategy to be aware of passing thoughts without reacting to them. Clearly, this takes some discipline and effort. Paradoxically, to adhere to this instruction well, it is also important to adopt a non-striving attitude toward the task. That is, wanting and trying too hard to achieve a certain “mindful state” can be counter-productive (Shapiro et al., 2018). Taking non-striving to another level, even the desire for relaxation, reduction of pain, or alteration of thoughts and emotions should be downplayed even if those are the reasons for initiating mindfulness practice (Baer, 2006). In sum, Shapiro et al. (p. 1697) describe non-striving in mindfulness practice as “an alert, relaxed attention (which requires effort to develop) without pursuing any specific goal,” and acknowledge that this attitude is often elusive. For example, the instruction not to strive hard in mindfulness practice may lead one to give up the practice too easily (Hopkins and Proeve, 2013). Suffice to say, non-striving is a nontrivial issue in the study of mindfulness that deserves more research as its coverage is currently limited in the extant literature.

Despite the centrality of non-striving in mindfulness, published academic work discussing non-striving in the context of mindfulness is surprisingly rare. By far, Shapiro et al. (2018) provided the most extensive discussion of non-striving in a narrative review which described paradoxes of mindfulness. In essence, Shapiro et al. (p. 1697) highlighted that practitioners often find it difficult to “… simultaneously allow what arise to arise – not strive to cultivate a particular state of mind – while trying to focus the mind on a particular object of attention…” They further explained that a way out of this paradoxical dilemma is to adopt the middle path, by striving at times and letting go in other instances. A more accurate interpretation would be to strive or to non-strive for different aspects of practice. For example, making the necessary effort to apply the mindfulness technique, while letting go of expectations. The difficulty of actualizing non-striving was documented in a research conducted by Solhaug et al. (2016), where they found that participants who underwent a mindfulness intervention program expressed lesser ease in appreciating the non-striving aspects of mindfulness compared to the attentional aspects. The former is somewhat counterintuitive while the latter is more concrete and less abstract. In essence, such empirical work on non-striving remains scarce. Further research is needed to better understand the operationalization of non-striving within mindfulness practices, particularly for future possible meaningful incorporations in clinical (Roemer and Orsillo, 2003) and other areas of interventions. Particularly, mindfulness practice tasks that can concretely introduce or elicit the experience of non-striving is especially needed.

Given the lack of research on non-striving and practice tasks that can introduce this concept well, the present study investigated the effects of a mindfulness-like practice task aimed at priming non-striving, futility, and process focus, on consequent non-striving behavior and performance. To this end, we created a brief intervention task – “Infinite Water Scooping Task,” which we speculate could be useful as a mindfulness practice for introducing non-striving in the future. This task involves using a small scoop to continuously transfer water over a string dividing a filled water container. Essentially, the task is perceptually futile in nature, as water would flow freely beneath the string after pouring, making the effort made in scooping and pouring seem purposeless. We speculate that performing this task would induce non-striving attitude temporarily even when practiced briefly for 10 min, compared to performing the control task of scooping and pouring from one container to another. A person performing the control task would see water level reducing in one container and increasing in another, whereas one performing the Infinite Water Scooping Task would not see incremental changes in water level over time. While both tasks involve essentially performing the same scooping and pouring action, it is intended the Infinite Water Scooping Task would elicit a mental state that departs from the usual outcome-oriented mental state that would be primed through the control task. The experience of performing the control task is deemed to be consistent with the *modus operandi* of most human as goal driven cognition is a norm in modern societies (e.g., Kenrick et al., 2010). On the other hand, the brief repetition of Infinite Water Scooping Task attempts to elicit non-striving orientation without explicitly instructing and emphasizing non-striving, as would be communicated during typical mindfulness practice.

The creation of the Infinite Water Scooping Task was inspired by the idea of strange loop proposed by Hofstadter (2007). Briefly, strange loop is characterized by self-referentiality and paradoxity. One example of strange loop offered by Hofstadter (2007) is M. C. Escher’s lithograph Drawing Hands, which depicts the right hand drawing the left hand, which in turn draws the right hand, forming an infinite loop. Another example is the case of Penrose stairs or impossible stairs created by Lionel Penrose and Roger Penrose, which is a continuously looping staircase that creates the impossible perception of climbing higher (Penrose and Penrose, 1958). In Hofstadter’s (2007, p. 101–102) words, strange loop is “... an abstract loop in which, in the series of stages that constitute the cycling-around, there is a shift from one level of abstraction (or structure) to another, which feels like an upwards movement in a hierarchy, and yet somehow the successive ‘upward’ shifts turn out to give rise to a closed cycle. That is, despite one’s sense of departing ever further from one’s origin, one winds up, to one’s shock, exactly where one had started out. In short, a strange loop is a paradoxical level-crossing feedback loop.”

While those above examples of strange loop comprised of optical illusions, the Infinite Water Scooping Task is aimed at eliciting a sense of paradox through a physical experience. In this task, when water is scooped and lifted across the string, there is an upward shift from one level of abstraction to another (where the abstraction refers to the state of the actions, such as where the scooped water is). The shift advances further when the water is poured out. Paradoxically, with this further advancement, water from the scoop merges with the original water source in the container, and the original state is revisited. In theory, this task can continue infinitely, like the continuously looping staircase in Penrose stairs. Hofstadter (2007) noted in the earlier description that some kind of “shock” is experienced in realizing the paradox of returning to the original state despite advancing. We liken this “shock” as the realization of the futility or the paradox of “physically exerting effort to scoop and pour water and yet effecting no change.” By continuously performing the Infinite Water Scooping Task for a few minutes, we expect participants to experience a temporary departure from the usual orientation of associating effort with outcome, which is typically ingrained in modern cultures.

We expect the experience of dissociation of effort with outcome through the Infinite Water Scooping Task to have some effects on subsequent non-striving behavior as performing this task continuously for a brief period could elicit psychological effects similar to mindfulness practice. The similarity lies in its repetitive nature, akin to that of, say, continuously paying attention to one’s breathing in mindfulness breathing exercises or repetitively watching how one walks in walking meditations. Typically, in mindfulness practice, one simply observes the continuous process unfolds cycle after cycle without expectation of any advancement. In other words, watching the in- and out-breaths, and appreciating that these are the only two states in the cycle, and that there is no need for striving to advance in breathing stages. In the Infinite Water Scooping Task, it is perceptually clear that there is no necessity of striving as there will be no visible change in water level. As the instruction to focus on the process is given concurrently, we therefore speculate that performing the task for 10 min is akin to mindful movement practice. Thus, upon completing this task, a mental state resembling mindfulness state could be activated. Here, we are primarily interested in examining whether there will be indications of weaker willingness to strive in a secondary task as a result of performing the Infinite Water Scooping Task.

In summary, we argued that non-striving is an important aspect of mindfulness practice, but it has not been sufficiently researched. To fill this gap in research, we conducted a randomized experiment to examine whether an essentially futile task designed based on the ideas of strange loop – Infinite Water Scoping Task – performed for 10 min has an effect on subsequent non-striving behavior and performance. Given the likelihood that the psychological experience of performing the Infinite Water Scooping Task is different from transferring water from one container to another (control condition), the main hypothesis tested is that the experimental group participants would spend less time in performing the subsequent word length comparison task due to lower efforts invested in counting carefully (i.e., showing lesser degrees of striving), relative to the control group.

## Materials and Methods

### Participants

Sixty participants comprised of 38 males (*M* age = 24.61, SD = 2.62) and 22 females (*M* age = 24.36, SD = 4.85) took part in the study. Participants were recruited from the university community *via* social media, posters, and word of mouth. The study was approved by the university’s institutional review board where the study was conducted. All participants provided informed consent. A SGD 10 (approx. USD 7.5) shopping voucher was given to each participant for his or her involvement in this study. They were randomly assigned to either the experimental or control condition in equal distribution. The methods were carried out in accordance to the guidelines stated by the university’s institutional review board.

### Brief Intervention Tasks

Common in the manipulation tasks of the experimental and control conditions was the task of scooping and pouring water at one’s self-selected pace for 10 min continuously using a small plastic scoop (15 cc in capacity with a 18-cm handle) in a standing position. The difference in conditions was in terms of the way the water container was set up on the table (i.e., using one or two containers). The video depicting the intervention tasks is provided in the Supplementary Material section. Before participants started the water scooping task, a brief condition-specific instruction was delivered to them *via* an Android tablet app in text and in audio script. When the time was up, the app played a chime to indicate the end of the intervention task.

#### Experimental Condition (Infinite Water Scooping Task)

In the experimental condition, a string was tied across the top of a container with the dimension of 42 (length) × 34.5 (width) × 17 (height) in cm, dividing the left and right of the container equally. The container was half-filled with tap water. Participants were tasked to scoop water from one side of the container, lifting the scoop over the string before pouring it back into the container. The task was meant to be perceptually futile and purposeless, because no drastic change to the water level would be observed as water flows across freely under the string within the same container. The instruction given to them *via* the app was to pour water using the scoop across the string at their own pace and to focus on the process during the task.

#### Control Condition

In the control condition, two containers, each with dimensions of 27 (length) × 20 (width) × 16.5 (height) cm were placed side by side. One of them was half-filled with tap water. Participants were tasked to scoop water from one container, and then pour it into the other container. The instruction given to them by the app was to pour water using the scoop across two containers at their own pace and that the task is practiced as a means of developing wrist control and strength. The control task was an appropriate match for the experimental task in terms of motor execution, effort, and timing. The effort applied was not futile and changes in water level can be observed. In contrast with the experimental task, the control task can be deemed as a common task.

### Secondary Task: Word Length Comparison Task

To detect the extent to which participants display non-striving behavior after undergoing the respective manipulations, we used a secondary task adapted from the task outlined by Touré-Tillery and Fishbach (2012) in their Experiment 5 and implemented it using OpenSesame (Mathôt et al., 2012) on a laptop computer. In this task, within each trial, two English words were shown separately on left and right sides of the screen. The task requires participants to choose the word with fewer letters by responding accordingly on the keyboard. There was no emphasis on timing and accuracy. The task is challenging as each trial had a pair of words that were different in length only by a letter. For example, “outstretches” vs. “repolarized,” “fishburger” vs. “assignation,” “birthweight” vs. “utterances,” etc. Words used were between 9 and 12 letters long, and were generated randomly using a computer script beforehand. Trials comprised of longer words would present more difficulty relative to trials with shorter words, by virtue of effort needed to count them.

The entire task comprised of two practice trials and seven blocks of six trials each (42 actual trials). We derived two types of measures from this task for each participant. First, the sum of correctly performed trials was calculated to serve as a measure of task performance. Second, the mean time (in ms) spent on each trial was calculated for all trials performed by each participant. We further derived the individualized mean time spent on trials that featured (1) 9 and 10 letters, (2) 10 and 11 letters, and (3) 11 and 12 letters, such that between-group comparisons can be made based on performances for trials of similar difficulty. This allows us to verify if the observed results were consistent regardless of task difficulty. The time taken to respond to the task was used as a proxy for striving as it reflects one’s effort to count the letters accurately before making a decision. Conversely, lesser time spent reflects non-striving as decisions were likely made without careful counting. As we did not emphasize on the speed or accuracy requirements, participants who strove to perform the task correctly might be more likely to be counting the words rather than estimating to get the correct answer. They were also told to move at their own pace and to feel free to take short breaks between blocks of trials if needed. In a nutshell, the salient instruction given to the participants was to pick the shorter word without time pressure. With that, we expected that the general speed-accuracy trade-off phenomenon predicted by Fitts’ law would naturally occur (Fitts, 1954), i.e., those who responded faster would tend to sacrifice accuracy, and vice versa. The experimental group, hypothesized to be non-striving, should respond faster but would have less correct trials, indicative of their lack of effort in getting the task right, if they simply adhered to the instruction of picking the shorter word without trying to be fast in responding.

### Procedure

Participants were individually tested in a quiet room and each session lasted for approximately 30 min. When participants arrived for the study, they were randomly assigned to one of the two conditions. After providing informed consent and given the shopping voucher, they proceeded to receive the task instructions and performed the water scooping task for 10 min according to the condition assigned, following which, they proceeded to complete the word length comparison task. Finally, participants were debriefed and thanked for their participation.

### Data Analysis

The initial step involved identifying and removing the outliers among the participants by detecting peculiar performances, such as those resulting from misinterpretation of task instructions. Next, we tabulated the descriptive statistics for sample-wise mean count of correctly identified trials and mean completion time spent. Data normality was examined using Shapiro-Wilk tests. Inferential statistical tests were performed using the robust Yuen’s *t* test for trimmed means, with bootstrapping set at 2,000, and trim level for the mean set as 0.20, as recommended in Field and Wilcox, 2017. Tests for group differences were conducted for the following dependent variables: number of correct trials (proxy for task performance), and mean time taken for completing each trial based on all 42 trials as well as that of trials with 9 and 10 letters, 10 and 11 letters, and 11 and 12 letters (proxies for non-striving to test the main hypothesis). The reason for analyzing the trials of similar word length separately is that shorter sets of words could take lesser striving/effort to perform while longer sets of words could require more effort, by virtue of total length presented. The additional analyses serve to determine whether group differences observed are present regardless of the word length used. Additionally, we also repeated the analyses for mean completion time of trials that were correctly performed to countercheck if the result was consistent with the earlier analyses based on all trials (correct and incorrect trials). The *yuenbt* function from R package WRS2 (Mair and Wilcox, 2015) was used for the robust statistical analysis. In this package, akp effect size which is a robust version of Cohen’s *d*, proposed by Algina et al. (2005), was used. The same rules of thumb as for Cohen’s *d* can be used to interpret the effect sizes; that is, 0.2, 0.5, and 0.8 correspond to small, medium, and large effects, respectively.

## Results

### Descriptive Statistics

Among the 60 participants, data from three participants were removed from further analysis as they achieved less than two correct trials in the word length comparison task. They failed to adhere to the given task instructions. This resulted in 27 participants in the control group and 30 participants in the experimental group. Of these remaining 57 participants, the mean count of correctly identified trials is 39.02 out of 42 (92.90%), and ranged from 34 to 42 trials. The mean trial completion time is 3,876 ms, and ranged from 786 to 10,770 ms. Results of Shapiro–Wilk test of normality for counts of correct response (*p* = 0.004) and time spent on each correct answer (*p* < 0.001) suggest that the distribution of the data is significantly different from normal distribution, i.e., normal distribution cannot be assumed. The decision to rely on robust approach for inferential statistical analysis based on Yuen’s modified *t* test for independent trimmed means with bootstrapping (Field and Wilcox, 2017) was thus made.

### Inferential Statistics

**Figure 1:**
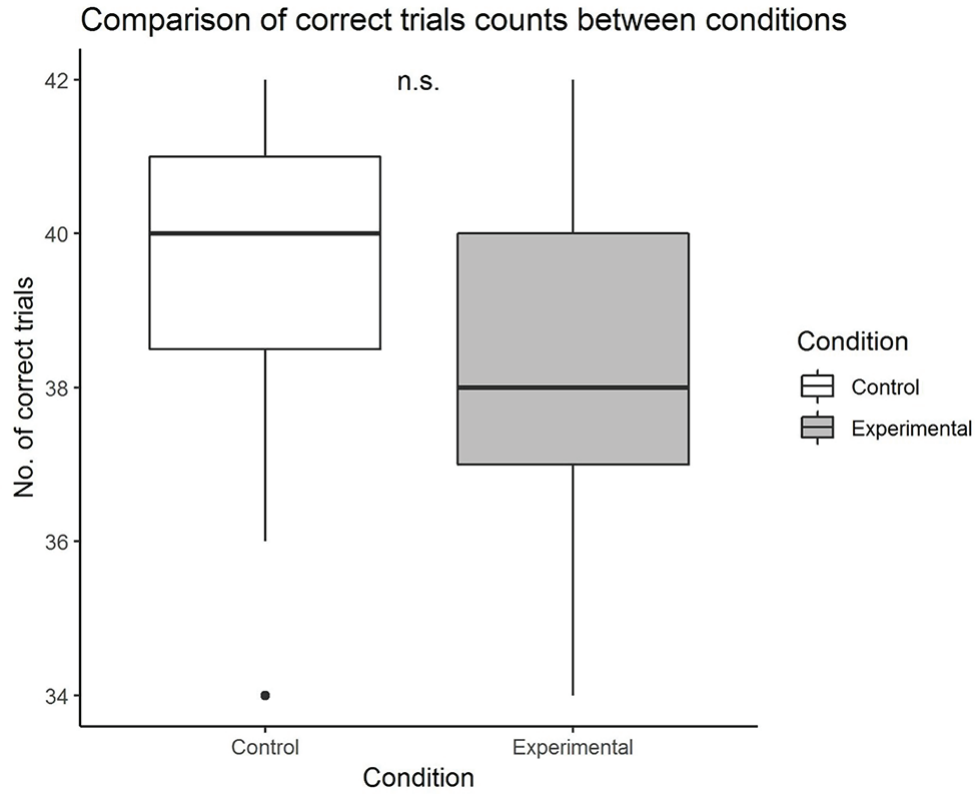
Comparison of number of correct trials between control and experimental groups.

Before testing the main hypothesis, we compared the number of correct trials between the two groups to ascertain if there was a difference in task performance (based on the number of correct trials). The robust Yuen’s *t* test for trimmed means (bootstrapping set at 2,000, and trim level for the mean as 0.20) detected no significant difference between trimmed means in number of correct trials for the control group and the experimental group, *M*_diff_ = 1.27 (−0.03, 2.56), *Y*_t_ = 2.02, *p* = 0.055. However, it can be interpreted that the value of *p* is close to the critical value for significance difference. Figure 1 suggests that the number of correct trials in the control group tended to be higher than that of the experimental group. The effect size was found to be 0.57, which was slightly larger than a medium effect size.

**Figure 2:**
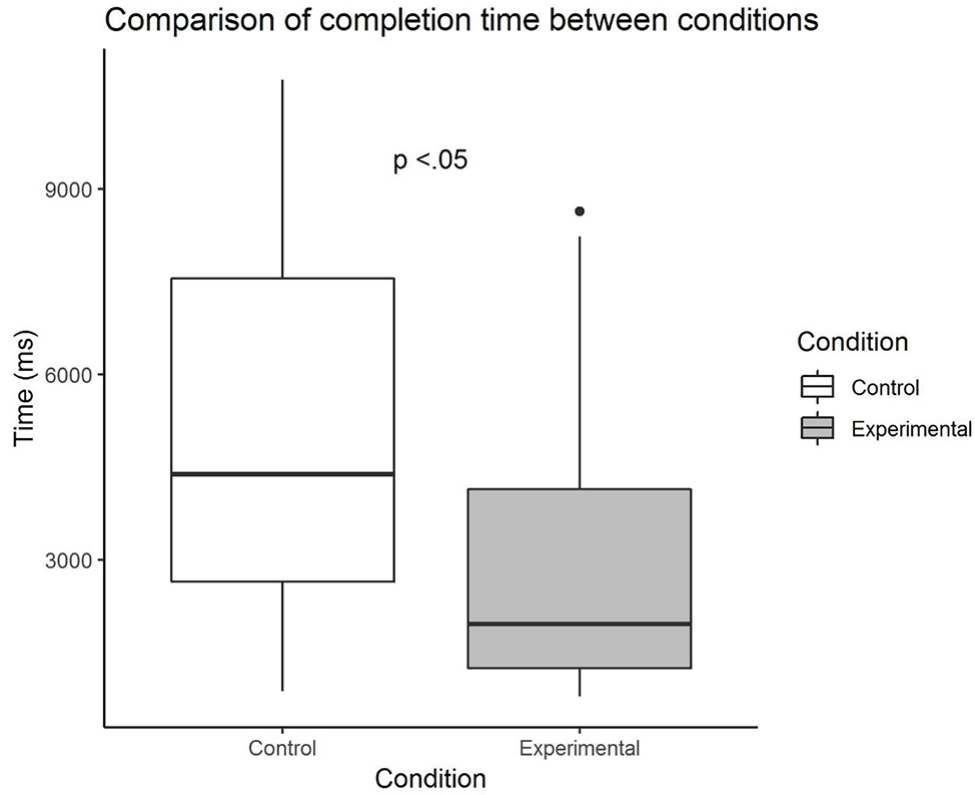
Comparison of mean time spent on each trial between control and experimental groups.

The main hypothesis that degree of non-striving differed between the two conditions was tested based on mean time spent on trials as a proxy measure for non-striving. When all 42 trials were considered, the robust Yuen’s *t* test for trimmed means (bootstrapping set at 2,000, and trim level for the mean as 0.20) showed that there was a significant group difference, *M*_diff_ = 2358.35 (573.48, 4143.21), *Y*_t_ = 2.65, *p* = 0.016. Figure 2 shows that participants in the experimental group spent significantly less time working on each trial compared to those in the control group. The effect size was found to be 0.78, which was close to a large effect size. The combination of results above shows that the control group took a longer time and yielded more correct trials, compared to the experimental group which took a shorter time but registered less correct trials. This suggests that the expected speed-accuracy trade-off occurred with the mere instruction to pick the shorter word without time pressure.

**Figure 3:**
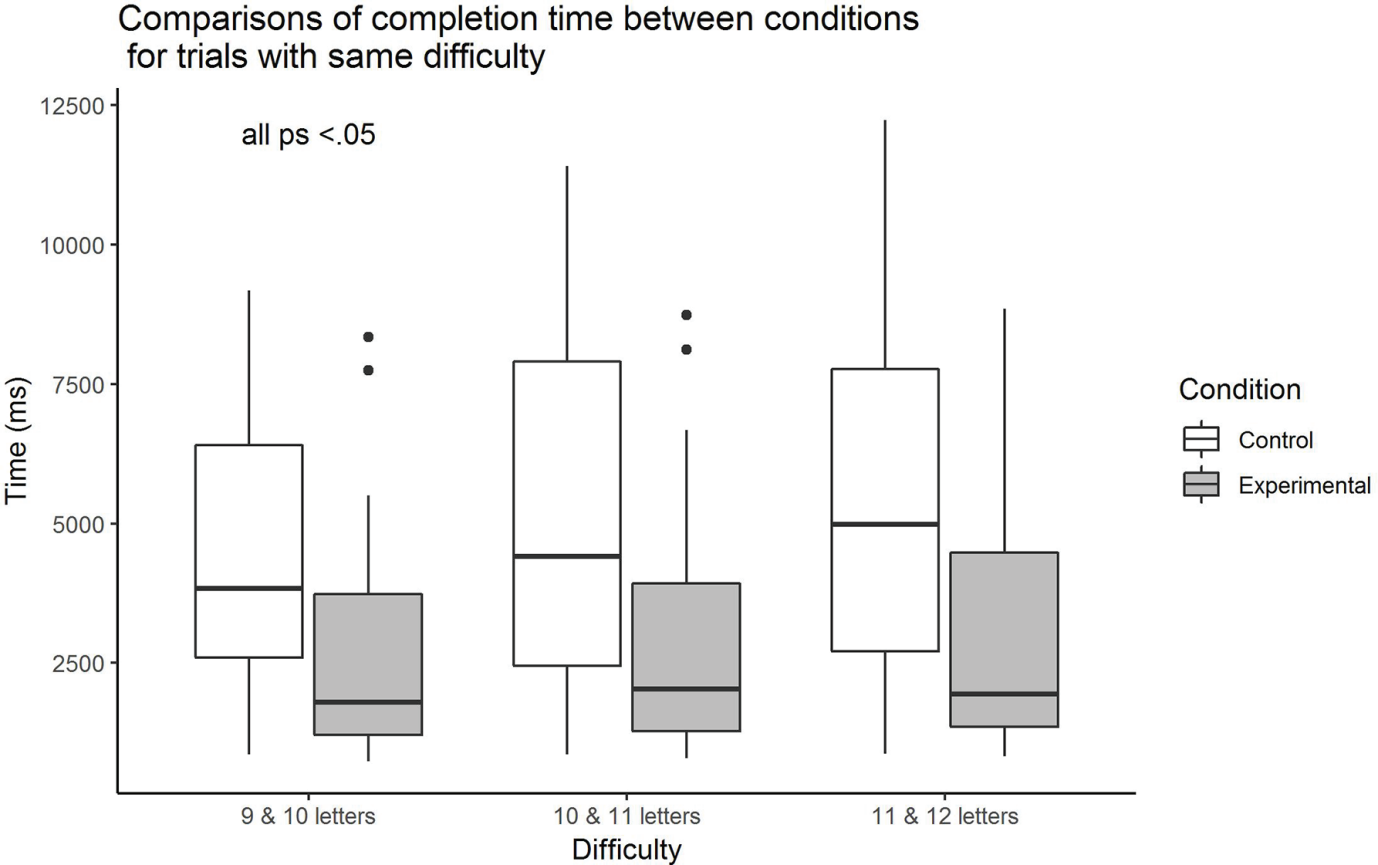
Comparison of mean time spent on each trial between control and experimental groups for three difficulty levels.

Further tests were undertaken to ascertain that the observed differences in time spent were also present for trials of similar difficulty (i.e., trials with words that had the same total number of letters). When trials with words of 9 and 10 letters are considered, the Yuen’s *t* test for trimmed means (bootstrapping set at 2,000, and trim level for the mean as 0.20) showed that there was a significant group difference, *M*_diff_ = 1871.91 (171.56, 3572.26), *Y*_t_ = 2.39, *p* = 0.031. The effect size of 0.70 was observed. For trials with words of 10 and 11 letters, the result again revealed a significant difference between groups, *M*_diff_ = 2378.98 (414.04, 4343.91), *Y*_t_ = 2.53, *p* = 0.017. The effect size was found to be 0.74. Lastly, for trials with words of 11 and 12 letters, the result is similar in that a significant difference between groups was observed, *M*_diff_ = 2693.13 (712.90, 4673.36), *Y*_t_ = 2.76, *p* = 0.010. The effect size value was large at 0.80. In all cases, the experimental group spent lesser time than the control group as depicted in Figure 3.

Lastly, as the analyses above were based on response timings of all trials, regardless of whether they were correctly or incorrectly performed, we repeated these analyses based on response timings of trials that were correctly performed to remove the possible noise in the data due to incorrect trials. Similar findings were observed. This additional observation suggests that the experimental effects are present even when we removed trials that were performed incorrectly due to the participants giving up or responding carelessly on certain trials. The effects of incorrect trials were negligible.

## Discussion

As there is a dearth of empirical research on the non-striving aspect of mindfulness, we tested the effects of a presumably futile strange loop task that has the potential of being developed as a mindfulness task, on subsequent non-striving behavior and performance. Although there was no significant group difference observed in terms of performance (based on total number of correct trials achieved), the overall results (close to significant *p* value and medium effect size) suggest that the control group showed tendencies of better performance than the experimental group. It is likely that those in the control group applied more effort and adopted the counting strategy to achieve the accuracy while the experimental group tended not to. Findings from the test of the main hypothesis suggest that participants who underwent the Infinite Water Scooping Task took significantly shorter time to perform the task compared to the control group. The effect was observed when all trials were considered, and when trials of similar difficulty level were considered. Collectively, our hypothesis that performing the futile strange loop task would prime subsequent non-striving behavior is supported.

The current outcome of eliciting non-striving behavior is noteworthy as non-striving is an important aspect of mindfulness practice that is rarely examined in research. Here, we observed that 10 min of repetitively scooping and pouring water over a string tied across a container, peppered only with a brief initial reminder to focus on the process of the actions (without mention of it being a mindfulness practice), led to non-striving consequently when compared to the control task. One speculation could be that the implicit realization of task’s futility or paradoxity arising from repetitive performance of the strange loop task (Hofstadter, 2007) is having an effect on one’s tolerance of ambiguity. Having to repeatedly scoop and pour water for 10 min without perceiving any sign of advancement may have gradually activated a sense of acceptance toward the ambiguities of the task. The inner dialogue could be something like: “this task is paradoxical in nature and does not make sense, but I continue with it nevertheless.” A sense of acceptance may have been resulted through repeated actions required of the task, akin to acceptance of unfoldment of in- and out-breaths in mindful breathing practices. Following the completion of this task, this tolerance for ambiguity or paradox, in turn, may have lowered one’s efforts to judge critically (or count carefully) during the subsequent task. Previously, Carson and Langer (2006) noted that actively thinking about paradoxes could increase one’s ability to tolerate ambiguity, which they viewed as a hallmark of mindfulness. In a way, performing the Infinite Water Scooping Task for a few minutes possibly succeeded in exposing the participants to a sense of paradox, akin to the sense of perceiving paradoxes when contemplating Zen koans (Christopher, 2003; Maex, 2011), which has been linked to psychological awakening.

In the present context, by psychological awakening, we mean leading participants out of their fixed ways of thinking or habits of striving, at least temporarily. Since the control group performed an objective-oriented task (i.e., clearly perceiving changes in water levels as a result of their action, and being told that the task is a means of developing wrist control and strength), performing the task could have heightened the sense of purpose-driven thinking and certainty that is implicitly habitual in most people (Covington, 2000; Monteiro et al., 2018; Ronkainen et al., 2018). Upon completing the control task that is perceptually more normal and non-ambiguous, the tendency to count the letters purposefully to achieve better accuracy in the subsequent word length comparison task may have been more readily evoked, as shown by the longer trial durations. Based on the shorter trial duration observed, it appears that those in the experimental group tended not to count before making a response. If they were striving to achieve task accuracy, they would have spent more time in the task by counting the letters carefully. As earlier alluded to, shorter time spent can be viewed as a sign of their willingness to tolerate ambiguity and errors in their task performance. They are striving less toward achieving accuracy as per required by the task. In a way, those in the experimental group may be less judgmental about themselves in terms of whether they performed the task accurately, while those in the control group seem more objective-oriented about their task performance. Taken together, the Infinite Water Scooping Task seems to be instrumental in leading participants out of their habits of striving, at least temporarily, when compared to the control task.

There are some practical implications arising from the current findings worthy of mention. First, given the current results, the Infinite Water Scooping Task could potentially be used to introduce the notion of non-striving within mindfulness practice in various contexts, such as when teaching mindfulness in clinical interventions (Roemer and Orsillo, 2003), in education (McKeering and Hwang, 2019), and in sport as part of mental skills training (Ortega and Wang, 2018). For instance, the Infinite Water Scooping Task can be used as an introductory task to teach what is meant by notions of repetition, focus on process, and purposelessness within mindfulness practice, relative to a task that is done with a purpose. Beginning mindfulness practitioners could especially benefit by experiencing the nuts and bolts of mindfulness practice through the physical nature of the task (Kee, in press), alleviating some of the difficulty raised by Solhaug et al. (2016). Second, the possibility of eliciting one’s tolerance for ambiguity and non-striving through the Infinite Water Scooping Task may be useful when it comes to helping one prepare for performance or creative situations when a creative, relaxed, and open mind-set is needed. For example, in sports, some athletes described their best performance during a flow experience (a peak psychological state in which one is fully immersed in an activity experience) as one of intense focus without making effort in keeping focused (Jackson and Roberts, 1992). The relaxed and non-striving experience accompanied by the Infinite Water Scooping Task or other similar tasks may help athletes navigate through the nuances accompanying such peak psychological states when they use it as part of their mental skills repertoires. For example, an archer can use this task to get him/herself mentally prepared to focus on the process and not the outcome for his/her shoot. Likewise, this task could add to the list of other known strategies for enhancing creativity such as one described and tested by Chirico et al. (2018), in that this task may help one to suspend judgment momentarily to allow creativity to flow. Lastly, beyond these two specific implications, there is potential for the mindful repetition of the Infinite Water Scooping task to be used as a mindfulness practice task for those who find difficulty in performing seated meditation, given that it is an overt task.

Although the findings and implications seem promising, there are some limitations that must be noted. First, as no manipulation check was conducted, we cannot be certain that the aforementioned non-striving or paradoxical effects were indeed evoked. One main reason for not conducting a manipulation check immediately after participants completed the brief intervention task is the concern that such check (e.g., using questions) may result in unexpected priming of psychological effects that affect the performance of secondary task. While we maintain that the paradoxity explanation is plausible for the experimental task given that the design principle of the Infinite Water Scooping Task took reference from concepts of strange loop (Hofstadter, 2007), we acknowledge that a better approach would be to conduct a manipulation check after the secondary task. Secondly, although we argued that the control task is aligned with participants’ *modus operandi* of being goal driven and performing the control task could be treated as typical experiences, the control condition can potentially prime goal-focused orientation, and thus the possibility of participant striving as a result cannot be ruled out. It would be worth considering including a neutral control task that does not prime striving and non-striving in future studies. Thirdly, although the main difference between the experimental and control condition is essentially pouring across a string within the same container compared to pouring across containers, the brief instruction of focusing on the process was given to the experimental group but not to the control group. This raises the question whether the observed effects came from the instruction to focus on the process. Although the likelihood is slim as the instruction was only given at the start in a brief fashion, future studies could consider keeping the instructions to focus on the process consistent for both groups to rule out this possibility. Fourthly, there could be an alternative interpretation in that those who underwent the Infinite Water Scooping Task actually strove harder than the control group, since they could have completed the word length comparison task faster due to them putting in more efforts to respond sooner without compromising on overall performance. It is a limitation in that there was no measure of perceived efforts. Nevertheless, since there was no explicit instruction to complete the task fast, we expected participants to direct their effort toward responding correctly as required by the task. The observed speed-accuracy trade-off supports this view. We maintain that the control group indeed made more efforts to count carefully compared to the experimental group, judging by the seemingly better task performance in the control group. In the future, potential mediators such as a perceived effort, drowsiness, sleepiness, and apathy can also be examined.

In summarizing the lessons learned in terms of designing and conducting this research on non-striving, we conclude that non-striving *per se* is very difficult to operationalize, which perhaps explains the lack of such works in the literature. The difficulty roots from the fact that, by definition, non-striving is the antithesis of striving. To understand non-striving without making reference to striving is not possible in some sense. Experimentally, it is difficult to create a neutral control task that is completely devoid of any form of striving to compare non-striving with. Any form of active control task would necessarily involve some level of striving, simply because there would be something to be done. That inevitably primes striving. On the other hand, adopting a passive task of sitting still as a control task is also not suitable as a control condition, because the non-striving condition such as the Infinite Water Scooping task would then be construed as requiring more striving than the control task. To this end, we propose a possible approach to make further inroads into understanding non-striving by focusing on the nature of the secondary task rather than on the manipulations. That is, by examining whether non-striving can manifest even in a seemingly effortless task. Putting it in the context of the present experimental setup, we can get closer to know if we effected non-striving if performance on word length comparison task involving very easy tasks (say, obviously long versus short words) is different for those undergoing control and experimental treatments. The assumption is that easy tasks take very minimal striving to perform. If the Infinite Water Scooping task resulted in even lesser striving for easy tasks, that could be a clearer indication of non-striving. Future studies could explore this approach to advance the operationalization of non-striving.

As mindfulness research advances over the years, many constructs related to mindfulness were examined to better understand mindfulness (Kee et al., 2019). Since works on non-striving have been especially scarce, the current study presents an initial foray into the empirical examination of non-striving in the context of mindfulness practice. The main takeaway message is that the Infinite Water Scooping Task seems instrumental in eliciting non-striving (relative to the control task), which may be useful for teaching mindfulness practitioners about the notion of non-striving. Clearly, more works in examining the nature and practical value of non-striving is warranted. Beyond investigating non-striving in the context of mindfulness, non-striving as a research topic is also relevant for line of research questioning the values of objectives and goals setting, such as works championed by Ordóñez et al. (2009) and Swann et al. (in press). There is a possibility that the line of research questioning the values of goals and non-striving through mindfulness may converge, hopefully illuminating further lessons beyond the duality of striving and non-striving. The conversations arising from the testing of Infinite Water Scooping Task may also contribute to discussions on altered sense of consciousness-related issues such as *wu-wei*, flow, mystical experiences, and awakening, since there had been discussion of “letting go,” non-striving, and such states in neuroscience literature (Austin, 2006, p. 275).

## Data Availability Statement

The datasets generated for this study are available on request to the corresponding author.

## Ethics Statement

The studies involving human participants were reviewed and approved by Nanyang Technological University – Institutional Review Board. The patients/participants provided their written informed consent to participate in this study.

## Author Contributions

YK, KA, and RF conceptualized and designed the study. KA and RF collected the data. YK and KA analyzed the data. YK, KA, and CL prepared the manuscript. All authors approved the final version of the manuscript for submission.

### Conflict of Interest

The authors declare that the research was conducted in the absence of any commercial or financial relationships that could be construed as a potential conflict of interest.

## Supplementary Material

The Supplementary Material for this article can be found online at: https://www.frontiersin.org/articles/10.3389/fpsyg.2019.02314/full#supplementary-material
